# The Mercurials Differentiated

**Published:** 1899-06

**Authors:** F. O. Pease

**Affiliations:** Chicago, Ill.


### THE MERCURIALS DIFFERENTIATED.

F. O. PEASE, M. D., CHICAGO, ILL.

From Gross' *Comparative Materia Medica* we find most of the following, comparing Mercury with Nitric Acid:

| MERCURY. | NITRIC ACID. |
|---|---|
| Light hair; skin and muscles lax. | Dark hair, rigid fibre. |
| Increased irritability of tissue. | Want of bodily irritability. |
| Pain passing outward. | Passing inward. |
| Rending pain downward. | Rending pain upward. |
| Sore pain in external parts. | Internal parts. |
| Eruptions generally dry. | Moist. |
| Scars get red. | Hurt with change of weather; break open. |
| Hot swelling of glands. | Painless (cold) swelling of glands. |
| Sweat smelling sour or moldy. | Sour or like urine. |
| Thirst predominates in all stages of fever. | Wanting in the chill. |
| Loss of hair: forehead and temples. | Top of head. |
| Moods serious; also amorousness. | Distrustful. |
| Absent-minded; imbecility; apoplexy. | Very rarely apoplexy. |
| Averse to greasy foods. | Desires fatty foods. |
| Diarrhœas usually painful. | Predominately painless. |
| Urging and tenesmus, during urination. | Urging and tenesmus after urinating. |
| Urine frequent, copious, hot, sour odor. | Scanty, cold or hot, smelling strong. |
| Menses late, profuse, flow bright red. | Too soon, blood dark. |
| Leucorrhœa either mild or acrid. | Rarely mild. |
| Expectoration during the day. | Worse at night or evening. |
| Foot sweat odorless. | Offensive. |
| Worse during and after sweat. | Better after sweat. |
| Worst at night when warm in bed in cold weather. | Much less aggravation in cold weather. |

Thus far and in many other conditions we find Nitric-acid is almost exactly the opposite of Mercury in the movement of its phenomena, which would seem to place this remedy not so much in an antidotal as an antipathic relation to Mercurius; therefore not a true homœopathic or dynamic, but more of a chemical antidote. However, in so far as the Nitric-acid provings show symptoms similar, or seem to work along parallel lines it may be a homœopathic similar to the mercurial disease.

It is interesting to note that Nit-ac. in potency is more effective in cases that have been abused by both Nitric-acid and Mercury. Susceptibility to both these drugs is an element that should be considered in prescribing either. The drug power (or "sick-making force") is marked in these two drugs. They profoundly affect the tissues and functions of the body, and we know also they are extensively abused.

A question which I believe is capable of being answered is suggested, How or why do potentized remedies act in accordance with the law of similars, and why are there so many so-called polychrests?

We are told it is because of more thorough provings, of better knowledge of them, and that these polychrests stand in closer relationship to the human organism than others. Very true, but why is it that among these very important polychrests there are so many drugs and substances that have for hundreds of years, and for generations past been used and abused by the antique school in their "system" of legendary therapia? Sulphur, Opium, Aloes, Mercury, Nucis Vomica, the Hemlocks, Antimony, Iodine, Potassium salts, Cinchona, etc., the old time "simples" and syrups, acids, alkalies, etc.

Are not these drugs polychrests simply because the abuse of them for centuries has caused the great artificial miasms or diseases to which they correspond? During these generations heredity, environment, and the abuses fostered by the therapeutic methods of the always dark ages of "regular" medicine have combined to burden humanity with corresponding artificial diseases. Hahnemann, by his discovery of potentiation, brought these drugs into curative relation to the diseases they had originally caused when given in crude and massive doses, and they become our invaluable polychrests. There is much evidence to prove this position.

Hahnemann, in *Lesser Writings*, p. 627, states in unmistakable terms as follows: "Every true medicine, namely, acts at all times, under all circumstances, on every living, animated body, and excites in it symptoms peculiar to it (even in a perceptible form if the dose be large enough), so that every living human organism must always and inevitably be affected by the medical disease and infected so to speak, which, as is well known, is not the case with respect to (medicines?)." The last word is evidently a typographical error. See the statement in preceding paragraph of the same article, viz.: "Diseases (spontaneous) are only the exceptional states of the human health," etc., therefore "medicines" above should read "spontaneous or natural diseases." At the top of the same page he says in italics, "The living organism is incomparably less capable of being affected by natural diseases than by medicines." I wish all would read the whole of this "spirit of the homœopathic doctrine of medicine" written by the master in 1813. It contains philosophy that becomes wonderfully bright and instructive to-day.

I mention a few of the points of difference in the mercurials that have proven valuable to me:

| MERC-VIVUS. | MERC-CORROS. |
| --- | --- |
| Weakness; obtuseness of memory. | Weakness of intellect. |
| Imaginary fears; tries to turn away; homesickness. | Hypochondriasis, discouragement. |
| Face pale, yellow, earthy, sunken, woe-begone, anxious. | Swelling and distortion of face; staring expression. |
| Teeth loose, fall out, caries, swollen, bleeding gums. | Loose teeth, gangrenous gums and tissues; burning pains. |
| Tongue, swollen, flabby; takes imprints of teeth; inflamed, indurated, suppurating; ranula. | Inflammation and swelling of buccal cavity, cheeks, gums, throat, soft palate. |

| MERC-VIVUS. | MERC-CORROS. |
| --- | --- |
| Much slime collects; coppery taste; ulceration of salivary glands. | Ptyalism; marked putrefaction, ulceration; disgusting odor from mouth. |
| Violent, burning thirst, especially for beer, milk, and liquid food. | Unquenchable thirst for cold water. |
| Merc-j-flav., right-sided throat affections predominate; Merc-j-rub., left side. | Tonsils enlarged; covered with ulcerations, either side or general. Common to both Merc-corr. and Merc-viv. |
| Merc-viv. stools, undigested, pitch-like, tenacious, dark-green and bloody mucus mixed. Urinary symptoms not nearly so marked *with* the rectal tenesmus as in Merc-corr. Urine more apt to be copious and frequent. | Stools yellow, green, bilious, *followed* by slime and blood; very marked tenesmus as in dysentery, associated with painful urinary urging; scanty or suppressed urine, passed in drops with great pain. |
| In diarrhœas, dysentery, etc., the "cannot get done" feeling may be considered as a general symptom of the mercurials—but more pronounced in Merc-viv. and Merc-dulcis. | In dysentery, without urinary tenesmus associated with the rectal tenesmus, this remedy would hardly be indicated. |
| Much blood in urine, presumably albuminuria is a symptom common to the mercurials. | Albuminuria, following scarlatina, and in Bright's disease. |
| Gonorrhœa, with phimosis or chancroidal ulcers; (Cup-sulph.) green discharges, worse at night. | Gonorrhœa, greenish, purulent, offensive; may be bloody, with filaments, flocks, or flesh-like shreds of mucus in the urine. Gonorrhœa complicated with tenesmus and painful diarrhœa, with the urinary group; or gonorrhœa with intensely inflamed, swelled external or internal piles, which may be or are ulcerating. |
| Merc-j-flav.: "Hard chancre, given at once will prevent secondary symptoms" (Hering). *Painless chancres,* with great swelling of inguinal glands, not disposed to suppurate. Merc- | Leucorrhœa, pale, yellow, disgusting, sweetish odor; acrid, profuse; expect the characteristic rectal and vesical tenesmus. |

| MERC-VIVUS. | MERC-CORROS. |
| --- | --- |
| j-rub.; suppurating buboes, ulcers in the bladder. | |
| OTHER MERCURIALS. | |
| Merc-j-flav., similar, except in odor, and has yellow leucorrhœa in children. | Fever; chilliness predominates; is worse from least motion (Nux) in open air in evening, especially the head at night. |
| Merc-j-flav., the open air relieves unpleasant sensations. | Sweat, often on the forehead, and general and cold, with anxiety. |
| Merc-viv. and others, worse in damp, cold weather (spring). | |
| Merc-dulcis, general cold, clammy sweat, smelling sour, of greasy consistency. | |
| Merc-j-flav., disturbed by frightful dreams, restless before midnight; Merc-j-rub., restless after midnight; dreams of swimming, traveling; lascivious. | Sleep, violent jerks of body on falling asleep. |
| Merc-j-flav., suppuration, even when acute inflammatory symptoms are not so marked as in Merc-corr. | Hot swelling of glands; inflamed buboes. |
| Disease movement not so rapid in the *Iodides* of Mercury as in the corrosivus. | Rapid progress of disease movement. |
| Merc-j-rub., fissures and cracks sore, oozing, and easily bleeding; pustules, inflamed base, scabs over, but pus oozes; ulcers inclined to pustulation. Follows Belladonna well in scarlatina, with the throat complications, especially left side. | Skin, ulceration tends to go deep; perforating and phagedenic. |
| Merc-j-flav., hard papules over body (Rhus-rad.) more tendency to scabbing and itching. | Purple spots; spots like scorbutus, mingled with itch-like eruption. |

In acute tonsilitis I have learned much of value in discriminating between the points of divergence in the five mercurials, viz.: Merc-viv., Merc-cyan., Merc-j-flav., Merc-j-rub., and Merc-cor. To generalize: Merc-viv. is related to the soft palate and tonsils, when ulcerated or swollen, and does its best work after we are sure there is pus formation; hastening the process (Hepar.) to pointing and discharge—*i. e.*, the disease movement is (*vide* Gross) from within outward—Merc-cyanuret. The tonsils are greatly inflamed, dusky red, swollen, with whitish spots, which soon become deep ulcers, with yellow green pus (diphtheria), great fetor, right side apt to be the worst, submaxillary glands swollen. Merc-j-flav.: We find the pharnyx, uvula, and tonsils inflamed, worse on right side; stiffness of jaws, cannot open mouth, voice altered (throaty), cervical glands enlarged, sensation of a lump in right side of base of tongue or throat, soreness extends up to right ear (left, Lac-can.), or even side of head and face. Tongue yellow, back part; clean in front; refuses to eat or drink.

Merc-j-rub.: The symptoms generally begin or are worse on the left side (Lac-can.); there is much hawking and spitting of tough white phlegm, submaxillary glands swollen, many ulcers in throat; tonsils more promptly suppurate, point, and discharge. Marked prostration; patches of membrane "looking like dried starch" (varnished or like the broken edge of china (Lac-can).

Merc-corrosivus: Either side may be attacked; there is more complaint of pain, less glandular swelling, and frequently there is marked urinary strangury, scantiness, or suppression, and prompt appearance of albumen in the urine.

To differentiate the mercurials from other remedies would be work for a much longer paper. In this, my desire has been, so far as possible, to aid in the separation of the mass of symptoms that are usually credited to the general term Mercurius, but which ought to be separated and placed to the credit of the particular preparation to which they belong. There are many remedies in our records of materia medica whose symptoms are almost hopelessly mixed—*e. g.*, the mercurials, Carbo-animalis and Carbo-veg., Arg-nit., Arg-met., Rhus-tox., Rhus-rad., etc., etc.

When homœopathic prescribers learn to differentiate closely between the medicinal members of the same family of drugs, there will be more enthusiastic and successful homœopathic prescribers.

I append a list of the mercurials, placing them in the order of study which seems to me would tend to further acquaintance with the genius of each drug in relation to the sick human organism. The list is: Merc-viv., Merc-corrosivus, Merc-sol., Merc-j-flav., Merc-j-rub., Merc-dulcis, Merc-præcipitatus, Merc-acetate, Cinnabaris, Red-vulcanite, Merc-sulph., and Amalgam. From a study of the Red-vulcanite and Amalgam we will gain most valuable knowledge of the diseases and symptoms which they cause, and by using them potentized, will be enabled to effect cures in many cases where we would miserably fail without them.

DISCUSSION.

Dr. Kennedy—I am glad that Dr. Pease has done this work. He has called attention to a fact that we all recognize —that there is too much generalization in regard to certain classes of remedies. I welcome it as a valuable aid to our study of the remedies. It will help us to get nearer the specific remedy.

Dr. Patch—I want to thank Dr. Pease, personally, for this paper, because I realize just how thankless such work often is. This is a great addition to our literature on this subject, and adds much to the value of our transactions.

